# Metaproteomics uncovers the functional capacity of a soil microbiome

**DOI:** 10.1038/s41598-026-47816-9

**Published:** 2026-04-17

**Authors:** Yuqian Gao, Joonhoon Kim, Ruonan Wu, Niaz Bahar Chowdhury, Joon-Yong Lee, Carrie D. Nicora, Ronald J. Moore, Matthew E. Monroe, Janet K. Jansson, Kristin E. Burnum-Johnson

**Affiliations:** 1 Earth and Biological Sciences Directorate, Pacific Northwest National Laboratory, Richland, WA USA; 2 Energy and Environment Directorate, Pacific Northwest National Laboratory, Richland, WA USA

**Keywords:** Metaproteomics, KEGG, MetaCyc, Soil microbiome, Functional annotation, Biotechnology, Computational biology and bioinformatics, Microbiology

## Abstract

The soil microbiome plays a vital role in key ecosystem processes, but its functional capacity remains poorly understood. Microbial activities underpin many applications in environmental biotechnology, such as nutrient cycling, contaminant degradation, and the recovery and transformation of minerals and elements. However, analyzing the complex soil metaproteome is challenging. Here, we propose an approach to explore soil metaproteomes, which will improve our understanding of the metabolic potential within the soil microbiome. As a proof of concept, we generated high-quality metaproteomes from native prairie soil using high-resolution tandem mass spectrometry. Over 15,000 peptides were identified using paired metagenomes. By using lowest common ancestor method, the peptides were conservatively assigned to 21 bacterial, fungal, and archaeal phyla or superphyla, including rare soil bacterial phyla such as *Candidatus* Tectomicrobia, as well as viruses. Functional analysis at the pathway level was performed using complementary KEGG and MetaCyc databases, revealing essential biogeochemical cycles, such as carbon and sulfur cycling. By combining taxonomic and functional analyses, we disentangled the relative contributions of individual soil microbial phylum-level taxon to community metabolic functions. This study highlights the importance of taxon-resolved functional analysis enabled by soil metaproteomics, surpassing the capabilities of other single-omics methods. It offers new insights into how individual microbes function within complex soil microbiomes, paving the way for more targeted microbial strategies to improve system performance in bioeconomy applications.

**Supplementary Information:**

The online version contains supplementary material available at 10.1038/s41598-026-47816-9.

## Introduction

The soil microbiome plays an important role in molecular transformations, providing significant potential for understanding nutrient cycles. Gaining a deeper understanding of these microbial processes could lead to the development of a wide range of bioproducts, including bio-based chemicals, specialized enzymes, pharmaceuticals, and biofertilizers. Furthermore, this knowledge can drive advancements in fields such as regenerative agriculture and methods for recovering and converting critical minerals and rare Earth elements^1–5^. This study aims to leverage the power of metaproteomics to uncover the functional profiles and identify taxonomic contributors within the soil microbiome responsible for carrying out distinct metabolic tasks.

Advances in metagenomic and metatranscriptomic sequencing have begun to unveil the functional potential of the soil microbiome. However, functional genes that are identified in metagenomes may originate from extracellular DNA from dead microbes or relic DNA, and thus overestimate the functional potential of the soil microbiome^6^. Metatranscriptomics can capture actively transcribed genes, yet its application to soils is often hindered by technical limitations. For instance, at least 80% of metatranscriptomic reads typically map to ribosomal RNA (rRNA) when rRNA is not depleted, while current rRNA depletion strategies remain suboptimal for complex soils and may introduce taxonomic and functional biases. Furthermore, metatranscriptomics cannot confirm protein translation or actual enzymatic activity. These challenges limit the ability of metatranscriptomics to provide comprehensive coverage of functional diversity in soil microbiomes. While extracellular proteins may contribute to similar uncertainties in metaproteomic data, similar to those posed by extracellular or relic DNA in metagenomics, the metaproteome represents a valuable approach to understanding the functional capacity of the soil microbiome and provides more direct evidence of realized metabolic processes than metagenomics and metatranscriptomics, as it captures proteins and enzymes directly involved in metabolic processes. It facilitates a closer connection between microbial functions and their ecological roles, offering an advantage in exploring microbial functions in complex environments. In addition, compared to metabolomics, which reflects transient end-products without clear taxonomic attribution, metaproteomics offers a more stable and mechanistic view of functional capacity.

Metaproteomics is increasingly recognized as a powerful tool for bridging the gap between genomic data and metabolic activity, providing deeper insights into microbiome functions. Global efforts, such as those led by the International Metaproteomics Initiative^7–9^, are advancing analytical methodologies and promoting collaboration within the microbiome research community. These efforts support the development of innovative approaches that align with the objectives of studies such as ours.

In addition to providing a comprehensive characterization of microbial diversity and functionality, metaproteomics offers a powerful tool for investigating specific microbial processes related to the solubility, cycling, transport, and transformation of nutrients, elements, and minerals within soil ecosystems. This capability opens new opportunities to directly study the membrane transporters, enzymes, and pathways that influence the bioavailability and fate of essential resources, supporting both fundamental research and practical applications for the bioeconomy and biomining^3,5^.

Despite its promise, current metaproteome studies face challenges in optimizing annotation methods and fully capturing the taxon-resolved pathway-level metabolic functions of the soil microbiome. To address these challenges, this study generated high-resolution metaproteomic datasets with paired metagenomic data to enable exploration of the functional capacity of the soil microbiome with a pathway-level perspective.

Fractionation is an effective method for deep metaproteomics analysis, particularly when working with complex environmental samples, as it allows for improved separation and detection of proteins by reducing sample complexity and increasing proteomic coverage^10–15^. Part of our datasets used in this study, specifically those from the 12-fraction scheme, come from a previous publication^16^. While our previous work focused on developing and comparing fractionation-based mass spectrometry methodologies for soil metaproteomic analysis, it did not explore the microbial or enzymatic functions within the Kansas native prairie soil microbiome. To address this gap, the present study builds upon our prior work by expanding the depth of metaproteomic data interpretation to investigate the functional potential of soil microbiome. Furthermore, this study enhanced the metaproteome depth and coverage by incorporating new samples processed through a 24-fractionation scheme, alongside those from the 12-fractionation scheme, enabling a more comprehensive investigation of microbial functions in soil ecosystems.

Additionally, this study demonstrates the power of using both KEGG^17^ and MetaCyc^18^ to gain a deeper understanding of molecular transformations in soil metaproteomic data. Combining KEGG and MetaCyc provides complementary insights, as KEGG focuses on pathway reconstruction and functional hierarchies, while MetaCyc offers detailed enzymatic reactions and curated pathway information, resulting in a more comprehensive analysis of microbial functions. By integrating these resources, our approach was able to identify the specific metabolic functions performed by soil microbiome members. This integrated analysis provides taxon-resolved pathway-level insights that not only deepen our understanding of complex soil microbiomes but also establish a robust analytical framework for future metaproteomic studies aimed at resolving taxonomy and functional potential.

## Material and methods

### Soil sample collection and preparation

Surface soils were collected from three field locations (soils A, B, and C) at the Konza Prairie Long-Term Ecological Research (LTER) field station in Kansas (see^19^ for the site map and soil content). The individual soil cores were excavated with a shovel to remove roots and large rocks and then homogenized manually. The samples were immediately frozen under liquid nitrogen, then shipped in a sterile packet on ice to the Pacific Northwest National Laboratory (PNNL). When received at PNNL, each sample was homogenized and sieved (4 mm) to remove roots and large rocks. Fifty-gram portions of soil were aliquoted into 50-ml Falcon tubes and flash-frozen at -80°C for subsequent analyses. Metaproteomic and metagenomic analyses were performed using aliquots of these samples without any additional perturbations or treatments. Specifically, metagenomic data and 24-fraction proteomic data were generated from pooled samples collected across three field locations (sites A, B, and C), while 12-fraction proteomic data were obtained from site A soil.

### Protein extraction, digestion, and peptide preparation

The detailed protein extraction and digestion methods were published previously^16,20^. The soil samples were quickly thawed and weighed into 10 g aliquots in 50 mL methanol/chloroform compatible tubes (Genesee Scientific, San Diego, CA) along with beads for cell lysis. The proteins were then extracted using the soil MPLEx method^21^. After methanol and chloroform extraction, protein pellets from both the interphase and debris were collected after being frozen and lyophilized for 2h. The protein pellets were solubilized by SDS-Tris buffer, mixed, and centrifuged to obtain the supernatant. The proteins were precipitated by adding up to 25% trichloroacetic acid and staying at −20°C overnight, and then washed with ice-cold acetone three times. The resulting protein pellets were solubilized by SDS-Tris buffer and digested using the Filter-Aided-Sample-Preparation (FASP) digestion method^22^. After digestion, salts were removed using a microspin C18 column (The Nest Group, Inc., Southborough, MA). Peptides from the aliquots of 10 g of soil were combined to generate a single peptide sample. A bicinchoninic acid (BCA) assay (Thermo Fisher Scientific, Waltham, MA) was performed to determine the peptide concentration.

### High-pH reversed-phase liquid chromatography fractionation

The peptide samples were first fractionated using a high pH reversed-phase liquid chromatography. Peptides were first loaded onto a Waters (Milford, MA) XBridge column (5 μm C18, 4.6 mm i.d. x 250 mm length). Then, fractionation was performed at 0.5 mL/min using an Agilent 1100 series HPLC system (Agilent Technologies, Santa Clara, CA) with mobile phases, A) 10 mM ammonium formate (pH 10.0) in water and B) 10 mM ammonium formate (pH 10.0) in 90% acetonitrile/water. The gradient profile (min, %B) was 0, 0; 10, 5; 70, 35; 85, 70; 95, 70; 105, 0; 120, 0. Fractions were collected every 1.25 min (96 fractions over the entire gradient) with either every 12th fraction combined for a total of 12 final fractions (rows of a 96 well plate was pooled) or every 24th fraction combined for a total of 24 final fractions (rows of a 96 well plate were pooled by every other row). All fractions were dried under vacuum and suspended in 25 μl water.

In the 12-fraction scheme, peptides (100 μg and 25 μg, respectively) from site A soil were loaded and fractionated using high pH reversed-phase liquid chromatography into 12 fractions, with triplicate technical replicates conducted. In the 24-fraction scheme, peptides were pooled from three field locations (sites A, B, and C soils) and fractionated into 24 fractions using the same high pH reversed-phase liquid chromatography as the 12-fraction scheme, with a single technical replicate performed. By pooling the samples and consolidating the findings, the goal was to capture conserved activities across the three soil sites rather than to compare individual site-specific activities. A total of 96 LC-MS/MS datasets were generated.

### Liquid chromatography-tandem mass spectrometry analysis

The liquid chromatography-tandem mass spectrometry (LC-MS/MS) analysis was performed using the Waters nano-ACQUITY nano UPLC system (Waters, Milford MA) with on-line trapping column and coupled with a Q-Executive HF mass spectrometer (Thermo Scientific, San Jose, CA). Analytical columns were packed in-house with 3 μm Jupiter C18 particles (Phenomenex, Torrence, CA) of 75 μm i. d. x 70 cm length. Trapping columns were packed in-house with 5 μm Jupiter C18 particles of 100 μm i. d. x 4 cm length. Mobile phase A was 0.1% formic acid in water, while mobile phase B was 0.1% formic acid in acetonitrile. The gradient profile (min, %B) was 0, 1; 2, 8; 20, 12; 75, 30; 97, 45; 100, 95; 110, 95; 115, 1; 150, 1. The ion transfer tube temperature and spray voltage were 325 °C and 2.3 kV, respectively. Data were collected for 100 min. Mass spectra were acquired from 400 to 2000 m/z at a resolution of 60 k (AGC target 3e6); while the top 12 (12 fraction set) or 10 (24 fraction set) precursors were selected for HCD MS/MS spectra acquired in data-dependent mode with an isolation window of 2.0 and at a resolution of 15 k (AGC target 1e5) using a normalized collision energy of 30 and either 45 s (12 fraction set) or 60 s (24 fraction set) dynamic exclusion time.

### Curated protein database construction, functional annotation, and taxonomic assignment

Paired-end shotgun metagenomes (HiSeq, 250-bp paired ends, Illumina, San Diego, CA, USA) and Moleculo long-hybrid metagenomes (Illumina TruSeq long-read hybrid subassembly) were generated from the same sites as described previously^23^. The resulting metagenome assemblies were used in this study and subjected to predict open reading frames (ORFs) that were translated into protein sequences using Prodigal (v2.6.3)^24^. These metagenome-derived protein sequences were used to construct a sample-specific protein database for downstream LC-MS/MS searches.

The protein sequences were queried against a range of functional annotation databases including EggNOG for annotating bacterial and archaeal proteins^25^ and the curated viral databases with previously published Hidden Markov models (HMMs)^26,27^. The search was performed using hmmsearch (Hmmer v3.1b2) with an E-value cutoff of 10^−5^. The bit scores of the qualified matches to each protein were ranked and the protein was annotated by the match with the highest bitscore. The same set of protein sequences was also annotated by the MetaCyc database (version 25.5)^28^ (blastp^29^, E-value < 10^−5^, bitscore > 50).

Taxonomy of the protein sequence was assigned based on the consensus taxonomy of the contigs where the protein was detected. A contig was considered taxonomically consistent only when at least two annotated ORFs of the contig shared the same taxonomic ranks. To achieve this, the contig taxonomy was annotated by CAT/BAT (v5.2.3)^30^ using the default parameters. The functionally and taxonomically annotated protein sequences predicted from metagenome assemblies were used as a curated database for searching the LC-MS/MS data.

### Peptide and protein identification from LC-MS/MS data

The LC-MS/MS spectra were searched using the MS-GF+ search engine (Sangtae Kim, 2014) against the protein databases described above. The search parameters were as follows: PrecursorMassTolerance, 15.0 ppm; IsotopeErrorRange, −1,1; TargetDecoyAnalysis, true; NumTolerableTermini, 2; MinPeptideLength, 6; MaxPeptideLength, 50; MinCharge, 2; MaxCharge, 5; and NumMatchesPerSpec, 1. The data of peptide-spectrum-matches (PSMs) was first filtered by a maximum of 2 missed trypsin cleavages and 0 irregular cleavages (NTT=2). The spectrum level peptide confidence score of the peptide-spectrum match (i.e., MSGFDB_SpecProb) and mass difference (in ppm) between the observed parent ion and the computed mass of the identified peptide (i.e., DelM_PPM in MS-GF+) values were optimized to achieve the highest number of peptide identification within each dataset while keeping the target-decoy-based false discovery rate (FDR) of both peptide identifications and spectrum identifications below 5%. The data was further filtered including only qualified peptides with at least 2 PSMs identified and excluding proteins with less than 1 qualified peptide identified. The adjusted mass error (DelM_PPM) was calculated based on the average of both the center of identified forward sequences and the optimized ppm cutoffs based on FDR.

To further support detection of proteins assigned to *Candidatus Tecomicrobia*, we employed a complementary validation approach. Proteins, that were assigned to *Candidatus Tecomicrobia* using the contig-based method described above, were searched against the clustered NCBI NR database, and taxonomy was re-evaluated using a lowest ancestor (LCA) method to the top ten best hits. This approach provided an additional layer of validation for the *Tectomicrobia* protein assignments.

### Viral peptide detection and classification

Beyond the standard LC-MS/MS spectra search setting, we applied additional conservative and virus-focused steps to minimize the detection of false positives. Specifically, we searched the spectra using MS-GF+ search engine against our published virus-specific databases that were curated from soil viral genomes and viral hallmark proteins from the NCBI Virus database, including structural and virion-associated proteins^31^. Peptide-spectrum matches were further examined and excluded if the matches had more than half the maximum mass difference and more than one-tenth of the maximum spectrum level peptide confidence scores of those matches identified in the non-virus-specific protein database generated from the hybrid metagenome. We retained the peptides that were only matched to the viral protein database but not to the proteins translated from metagenome assemblies. The matched peptides with more than 2 missed trypsin cleavages or more than 1 irregular cleavage were also excluded. Additionally, we performed filtering to exclude any spectra that were matched to non-viral proteins in the metagenome. The putative viral peptides were manually inspected to remove the ones with low MS/MS fragmentation coverage (<30%) and low MS peak quality (no chromatography peaks of MS1). A viral protein with at least two viral peptide matches was considered a positive detection. A viral contig with more than two proteins detected was retained for clustering and classification analysis.

A sanity check of the detected viral contigs was performed using checkV^32^ to remove any attached host sequences. The resulting viral contigs were clustered with Viral RefSeq genomes (v201) based on a scored protein-sharing matrix implemented in vConTACT using default parameters^33^. The clusters generated by vConTACT were visualized as a network, with the clustered viral contigs as nodes and the calculated node connectivity as edges. Unclustered viral contigs were searched against the NCBI virus database^34^ based on sequence similarity, and the top ten hits were retained. Viral contigs were considered a close match to NCBI reference viruses if 1) the E-value was lower than 10^−10 and the percent of identity was greater than 75%, or 2) the average nucleotide identity and percent coverage exceeded 95% and 85%, respectively^35^. Additional clusters of viral contigs and NCBI reference viruses were merged into the vConTACT clustering network. The size of the nodes in the merged network represented the number of proteins detected per viral contig. The taxonomy of the clusters was assigned based on the annotations of the clustered reference viral genomes.

### Pathway analysis using KEGG and MetaCyc

KEGG orthology (KO) and KEGG pathways (release 112.0) were downloaded using KEGG API^36^. The proteins matched by the quality-filtered spectra were annotated with KO and then assigned to the respective KEGG pathways. The annotated spectra were included in the following calculations of spectra count. The total number of spectra mapped to KO class or KEGG pathway was calculated for each phylum. If spectra were mapped to multiple KO or pathway entries, we applied a proportional counting strategy: counts were divided evenly across all mapped entries. This approach preserves the total spectral evidence, prevents over-counting of conserved peptides that map to multiple homologs, and provides an unbiased way to distribute ambiguous peptide matches among equally plausible assignments. Visualization on KEGG pathway maps was performed using KEGG markup language (KGML) and Biopython version 1.79^37^.

Similarly, the same set of proteins matched by the quality-filtered spectra were annotated with MetaCyc and categorized into MetaCyc pathway classes and instances using Pathway Tools version 25.5^38^. A multi-organism pathway/genome database was created for the soil microbiome by providing MetaCyc identifiers directly and using a pathway prediction score cutoff of 0, while disabling name matching, taxonomic pruning, or optional refinement tasks to avoid inferring erroneous reactions. The total number of spectra mapped to MetaCyc pathway class or instance was calculated for each phylum. If spectra were mapped to multiple pathway entries, counts were divided by the total number of mapped entries. Visualization on MetaCyc pathway maps was performed using Pathway Collages^39^.

Relative contributions of different soil bacterial phyla to the annotated KEGG and MetaCyc pathways were calculated by normalizing the abundance of the peptides by the sum across all pathways and phyla. For visualization, the 15 most abundant soil bacterial phyla and up to 25 most abundant metabolic pathways were shown. Dot plots were generated using seaborn package version 0.13^40^.

## Results & discussion

### Functional composition of soil microbiome

Soil samples were collected from the Konza Prairie Biological Station (KPBS) in Kansas^16^. We generated high quality soil metaproteomic data that allow for the detection of microbes and their metabolic functions. Total proteins were extracted and analyzed using high-resolution tandem mass spectrometry with offline two-dimensional orthogonal liquid chromatography separations (2D-LC-MS/MS), performed on 24 and 12 fractions obtained from pooled soil samples that combined material collected from multiple sites in Kansas. We pooled samples from three Konza Prairie LTER locations to focus on conserved enzymatic activities, a strategy supported by prior findings that 94% of unique Enzyme Commission (EC) numbers were shared among these sites with less than 1% unique to any single location^23^. After searching against the paired proteome database, a total of 15,238 peptides and 70,185 peptide-spectrum-matches (PSMs) were identified with over 98% PSMs having mass error less than 5 ppm and MSGF+ spectral probability score below 1.3E-10. These peptides represented 11,125 proteins and 9,086 contigs (Supplementary Table 1). The PSMs are calculated to be 1.9% of the 3,716,352 total MS2 spectra acquired, which is more than a 70% increase in MS/MS spectra annotation rate (the ratio of PSM to the total number of MS/MS (MS2) spectra) from the previous publication using only the 12-fraction datasets^16^. On average, the 24-fraction sample compared to 12-fraction samples improved protein identification by ~20%, peptide detection by ~70%, and Peptide-Spectrum Matches (PSMs) by ~100%. These results highlight the efficiency gains achieved with more fractions (Supplementary Figure 1). Most of the identified PSMs were assigned to Bacteria, accounting for 94% of the total identified PSMs (Supplementary Figure 2). Among the bacterial phyla, the six with the most PSMs were Proteobacteria, Acidobacteria, Actinobacteria, Verrucomicrobia, *Candidatus* Rokubacteria, and *Candidatus* Tectomicrobia (Supplementary Figure 3). The higher PSM counts in these phyla suggest these groups may have greater relative protein abundance and/or higher gene expression, indicating their significant contribution within the microbial community.

**Fig. 1:**
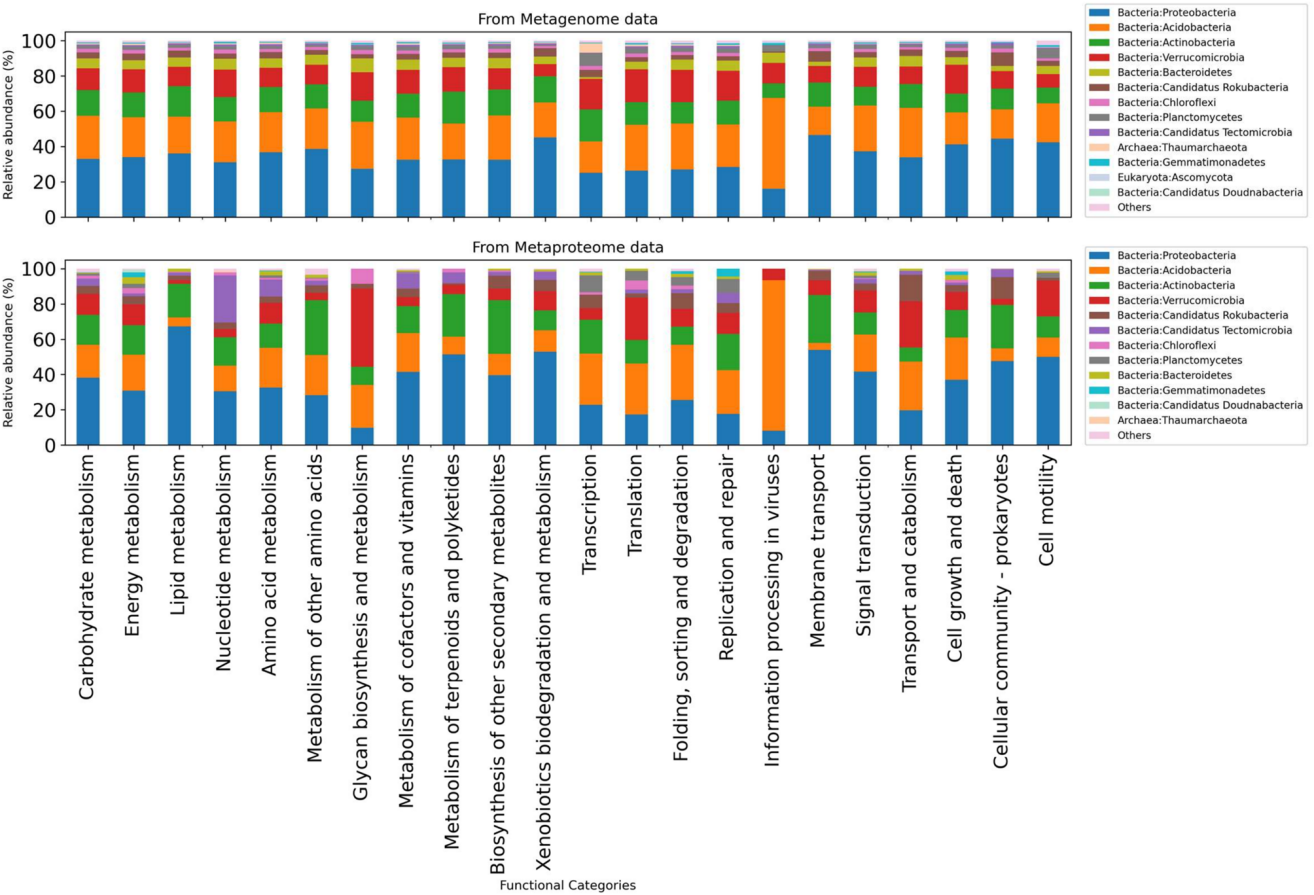
Stacked bar charts comparing the relative abundance of different taxa across various functional categories derived from metagenomes (top) and metaproteomes (bottom), respectively. In the metagenome, the y-axis is based on the number of proteins, while in the metaproteome, it is based on the number of peptide-spectrum matches (PSMs). Only the top taxa contributing at least 1% to the metagenomes or metaproteomes were highlighted, with the rest grouped as Others.

The distribution of metabolic pathways identified in the paired metaproteome and metagenome was highly consistent (Supplementary Figure 4). While metagenomics covers the entire microbial community and highlights their potential functions, metaproteomics provides insight into the specific organisms carrying out functions within the microbial community (Fig. 1). This underscores the complementary nature of these approaches and their combined utility for exploring microbial ecology. When comparing protein levels to genomic data, Proteobacteria appear to have an increased role in lipid metabolism, suggesting their increased activity in energy storage and membrane dynamics. Similarly, *Candidatus* Tectomicrobia shows an increased role in nucleotide metabolism, potentially reflecting their importance in genetic material processing and repair, and Verrucomicrobia, on the other hand, shows enriched activity in glycan biosynthesis and metabolism, highlighting their role in carbohydrate-related processes crucial to soil ecology.

### Underrepresented soil microbes

Using the deep soil metaproteome, we verified the presence of some underrepresented soil microbes. Among these was *Candidatus* Tectomicrobia, a recently discovered uncultured bacterial phylum^41^ that is commonly associated with marine sponges. Despite its prevalence in aquatic ecosystems, very little is known about its presence in soil^42^. In this study, we identified a total of 616 highly specific PSMs representing 136 unique peptides and 64 proteins unique to *Candidatus* Tectomicrobia (Supplementary Table 2). These findings confirm the active presence of Tectomicrobia in Kansas native prairie soil.

**Fig. 2:**
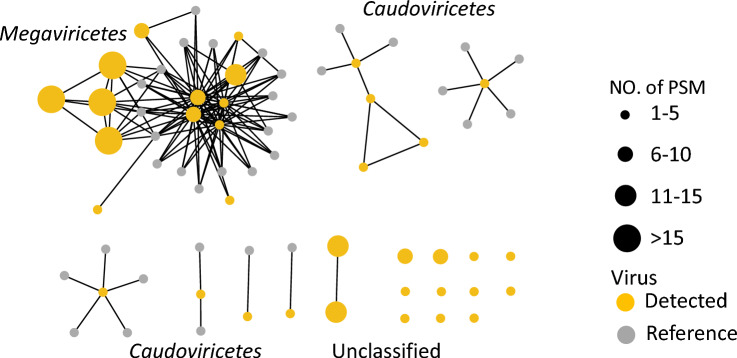
Viral clusters detected in the Kansas native prairie soil metaproteome. The detected viruses (nodes in yellow) and the clustered reference viruses (nodes in grey) are visualized in a network. The size of the nodes represents the number of peptide-spectra-matches (PSMs) per virus detected in the Kansas native prairie soil metaproteome dataset. The taxonomic assignments were labeled by the side of the viral clusters.

We also detected PSMs that only matched the curated viral proteins, resulting in a detection of 77 proteins from 34 unique viral contigs in the soil metaproteome (Fig. 2, Supplementary Table 3). The viruses detected were classified as *Megaviricetes* and *Caudoviricetes*. *Caudoviricetes* are known as the dominant DNA viruses infecting bacteria in soil^43^. Their relatively high abundance may explain the high coverage observed in metaproteomes. Four viruses with more than 15 proteins each were classified as *Megaviricetes* (Fig. 2). *Megaviricetes* are known to include giant viruses with genome sizes up to several megabases, comparable to or even larger than some microscopic cellular organisms^44^. The larger genomes of *Megaviricetes* may lead to a greater diversity of encoded proteins, increasing the chance of detecting their peptides in the metaproteome data compared to other viruses. Detection of viral proteins in soil metaproteomes remains an emerging area with limited benchmarking, so we interpret these identifications conservatively, and targeted validation will further strengthen confidence in complex samples.

### Improved annotation using paired metagenomics vs. using a conventional approach

The quality-filtered MS data are often searched against large, publicly available databases, such as the entire UniProt bacteria database, which contains an extensive and non-site-specific collection of proteins. While comprehensive, this approach demands significant computing time and can lead to an increased false discovery rate (FDR). In contrast, we found that searching the MS data against proteins predicted from a soil metagenome sequenced from the same site^23^ provided improved results. Specifically, this approach led to more peptide identifications and peptide-spectrum matches (PSMs) (Supplementary Figure 5), demonstrating the superiority of a customized database tailored to the local microbial community. This observation aligns with findings from other metaproteomics studies, which emphasize the advantages of a customized database for peptide detection and protein inference^45,46^.

To evaluate the effectiveness of the paired metaproteome, we compared this approach with annotations using the entire UniProt bacteria database, which contains 40744608 proteins (dereplicated at a 50% threshold identity). When searching against the UniProt database, a total of 2,494 peptides and 10,046 PSMs were identified. In contrast, using paired metagenomes, we annotated approximately six times more unique peptides than with the public database, despite the metagenome-informed protein database being fifty times smaller. This improvement demonstrates the power of leveraging site-specific metagenomic data for metaproteomic analyses.

### Function partitions of soil microbes

**Fig. 3:**
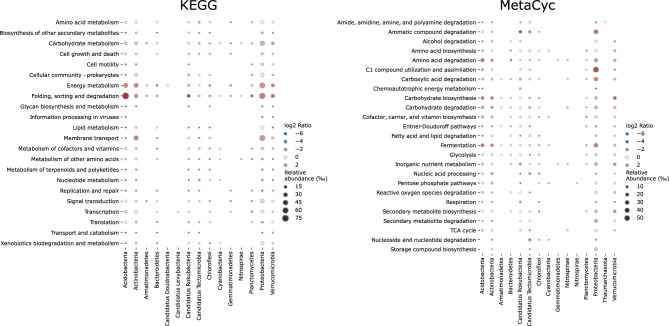
Functional annotation of soil metaproteomic data using KEGG and MetaCyc databases. The size of the circles indicates the relative contributions of each of the 15 most abundant soil bacterial phyla to different metabolic pathways. The relative contribution per metabolic pathway was calculated by the abundance of the peptides per phylum relative to all detected phyla. The color of the circles indicates the relative contribution of a metabolic pathway for each of the 15 most abundant phyla. The relative pathway contribution was calculated by a log2 ratio of abundance for a metabolic pathway in the metaproteomes to the mean abundance across all metabolic pathways within each phylum.

The metaproteome was annotated using two comprehensive reference databases, KEGG^36^ and MetaCyc^28^. Of the 11,125 identified proteins, 8,100 (72.8 %) were mapped to over 808 molecular-level KEGG Orthology (KO) functions and assigned to 147 metabolic pathways (Fig. 3, Supplementary Table 1), while 7,702 (69.2 %) were mapped to the MetaCyc database, which corresponded to 851 unique entries and 460 metabolic pathways in MetaCyc (Fig. 3, Supplementary Table 4). We systematically compared the annotated pathways in KEGG and MetaCyc based on the similarity of Enzyme Commission numbers (Supplementary Table 5). At the pathway class level, 10 pathway classes such as carbohydrate metabolism and amino acid metabolism had a significant overlap (Jaccard similarity > 0.2), and they were consistently annotated by both KEGG (out of 22) and MetaCyc (out of 51). However, the rest of the pathways were annotated by only one database, such as folding, sorting, and degradation in KEGG and chemoautotrophic energy metabolism in MetaCyc. This demonstrates the complementary benefit of combining KEGG and MetaCyc results for a more comprehensive functional annotation of the soil metaproteome^47^.

**Fig. 4:**
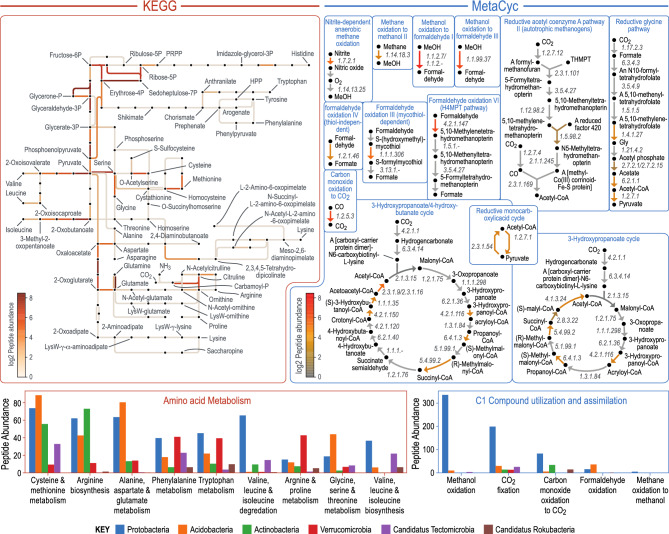
Soil microbiome peptide abundances visualized on KEGG and MetaCyc pathway maps. The color of lines in pathway maps represents the log2 peptide abundance from all phyla for different metabolic functions involved in amino acid biosynthesis (KEGG) and C1 compound utilization (MetaCyc). The bar graphs show the contributions from the six most abundant soil bacterial phyla to specific metabolic functions.

We used a curated, metagenome-informed protein database with both taxonomic and functional annotations to analyze the soil metaproteome, demonstrating the functional contributions of individual soil phyla (Fig. 3). Most metabolic pathways were detected in multiple bacterial phyla, highlighting shared functional potentials within the soil microbiome. However, the relative contribution of the individual taxon to specific metabolisms varied. This functional redundancy is a key feature of the soil microbiome, supporting its resilience and resistance to environmental disturbances^48^. We further visualized soil microbes’ contributions to specific metabolisms using pathway maps from KEGG and MetaCyc (Fig. 4). Initially, we used KEGG to show how each phylum contributed to the metabolism of the 20 essential amino acids, which play vital roles in carbon, nitrogen, and sulfur cycles. *Candidatus* Tecomicrobia showed significant involvement in branched-chain amino acid biosynthesis and degradation (KEGG pathways ko00280 and ko00290), while Verrucomicrobia were relatively more capable of arginine and proline metabolism (KEGG pathway ko00330), including proline biosynthesis and degradation as well as polyamine biosynthesis. Next, we used MetaCyc’s ability to generate customized pathway maps to illustrate how different soil microbes contribute to C1 compound assimilation and utilization. MetaCyc features multiple pathway variants (e.g., five methanol oxidation and seven formaldehyde oxidation pathways) and recognizes taxa that possess them, offering new insights into modular methylotrophy^49^. For example, the abundant Proteobacteria mainly participated in CO_2_ fixation and methanol oxidation, aligning with previous studies^50^. Conversely, Acidobacteria made the largest contribution to formaldehyde oxidation, consistent with earlier soil metaproteomics research^51^. This information highlights the roles of these microbes in carbon and energy cycling, providing new opportunities for mining microbial taxa with traits of interest, which could be useful for environmental and industrial applications.

**Fig. 5:**
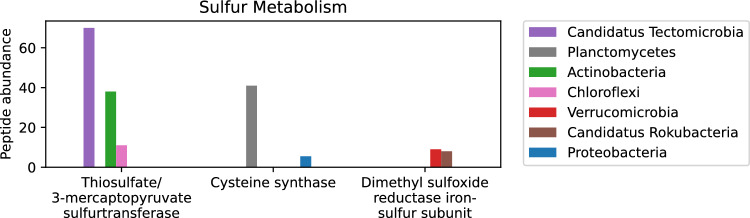
Soil microbiome peptide abundances in the KEGG sulfur metabolism pathway. The bar graphs show the contributions from classified soil bacterial phyla to specific metabolic enzymes, including K00184 (dmsB): dimethyl sulfoxide reductase iron-sulfur subunit [EC:1.8.5.3], K01011 (TST, MPST, sseA): thiosulfate/3-mercaptopyruvate sulfurtransferase [EC:2.8.1.1; EC:2.8.1.2], and K01738 (cysK): cysteine synthase [EC:2.5.1.47].

Finally, we explored the enzymes involved in sulfur metabolism (KEGG pathway ko00920), which can contribute to the recovery of critical minerals and bioremediation (Fig. 5; Supplementary Figs. 6-8**; **Supplementary Table 6**)**. Thiosulfate/3-mercaptopyruvate sulfurtransferase, involved in the detoxification of cyanide, was the most abundant enzyme in sulfur metabolism with large contributions from *Candidatus* Tecomicrobia and Actinobacteria. The second most abundant enzyme was cysteine synthase, which facilitates the incorporation of inorganic sulfur from the environment into organic molecules, mostly contributed by Planctomycetes. Dimethyl sulfoxide reductase was also observed from Verrucomicrobia and *Candidatus* Rokubacteria, which plays an important role in anaerobic respiration and biogeochemical cycles^52^. Dimethyl sulfoxide reductase and other sulfur-oxidizing enzymes can be employed to transform sulfide into sulfate, reducing the toxicity of sulfur-rich environments^52^. Abundant sulfur in geological environments can be a factor in creating the chemical conditions necessary for the formation and concentration of many valuable metal deposits. Sulfur-rich environments are therefore considered prime targets for the exploration and extraction of critical minerals^53^.

## Conclusion

Here, we demonstrated an approach using soil metaproteomics to characterize the functional capacity of the soil microbiome by leveraging paired long-read metagenomic data and annotating with complementary functional databases such as KEGG and MetaCyc. This method provides insights at multiple levels, including taxonomic diversity across functional categories, coverage of rare species, pathway information from KEGG and MetaCyc, and details down to the level of individual enzymes. These analyses offer new insights into the functional roles of different soil bacterial phyla and enzymes in key processes such as the biogeochemical cycles of carbon and critical minerals. Importantly, the recovery of minerals from metal mines could reduce reliance on imports for critical minerals^54^, and soil metaproteomics could aid in identifying microbial pathways involved in mineral cycling and recovery. With advancements in mass spectrometry instrumentation and data-independent acquisition, soil metaproteome studies will reach even broader coverage in the future^55^. The approach presented here provides an innovative workflow for future in-depth soil metaproteomic research. For instance, future efforts could combine metaproteomics with genome mining to validate the expression of biosynthetic gene clusters, such as those encoding ribosomally synthesized and post-translationally modified peptides (RiPPs) and other peptide-based natural products^56^, opening new avenues in the discovery of bioactive compounds.

## Supplementary Information

Supplementary Information 1.

Supplementary Information 2.

Supplementary Information 3.

## Data Availability

The MS proteomics data has been deposited to the ProteomeXchange Consortium via the MassIVE partner repository with the accession number MSV000090821, and the data can be accessed through the MassIVE website at https://massive.ucsd.edu. The metagenome assemblies were available at NCBI under Bioproject PRJNA320198^23^.
