# Supplementary material for: Metaproteomics uncovers the functional capacity of a soil microbiome
Source: Sci Rep. 2026 Apr 17;16:22142. doi: 10.1038/s41598-026-47816-9 (PMC13370013; doi:10.1038/s41598-026-47816-9)
Supplement: Supplementary file 1 — Supplementary Information 1. [file 41598_2026_47816_MOESM1_ESM.pptx]

## Slide 1
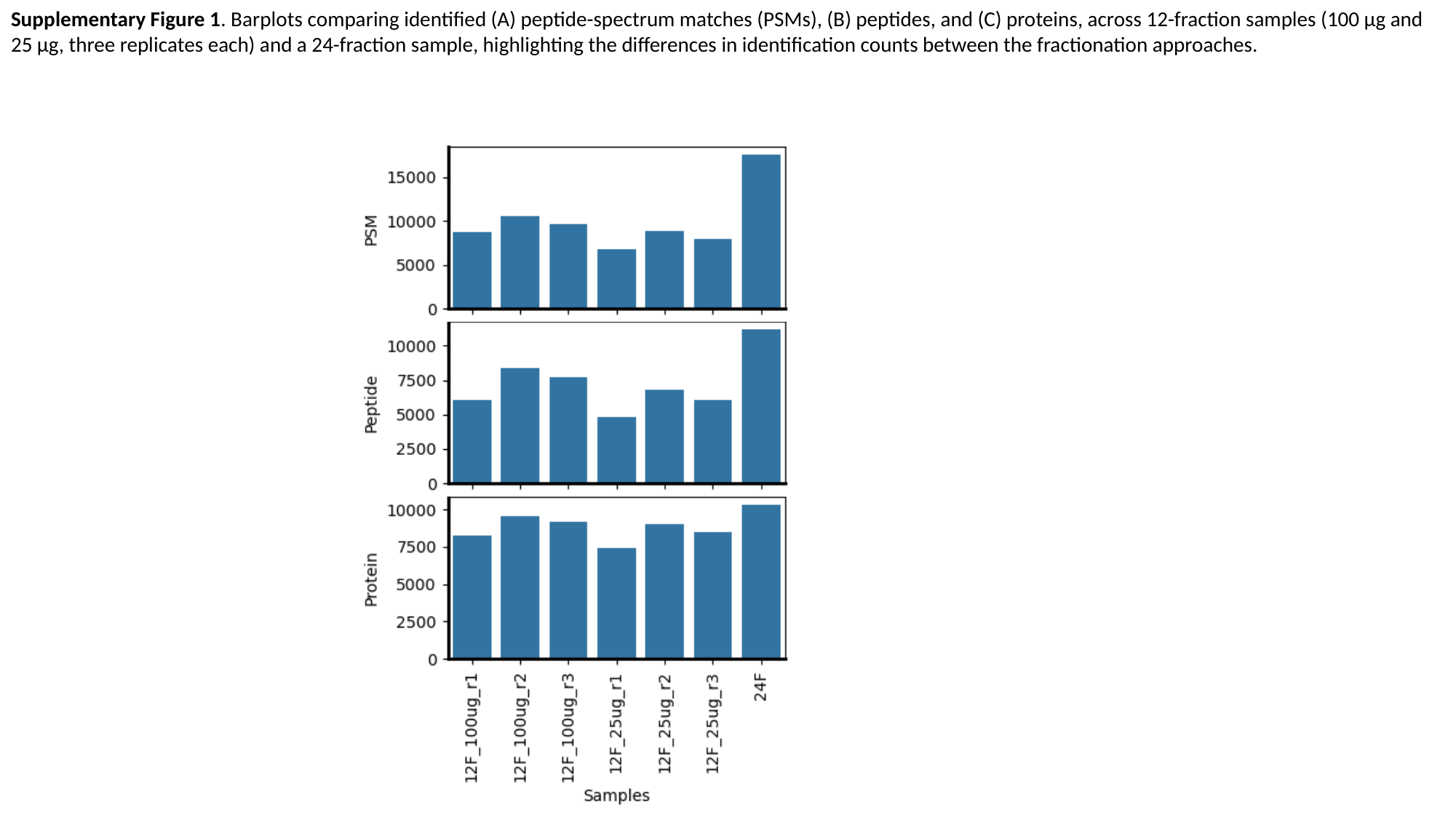

Supplementary Figure 1. Barplots comparing identified (A) peptide-spectrum matches (PSMs), (B) peptides, and (C) proteins, across 12-fraction samples (100 µg and 25 µg, three replicates each) and a 24-fraction sample, highlighting the differences in identification counts between the fractionation approaches.

## Slide 2
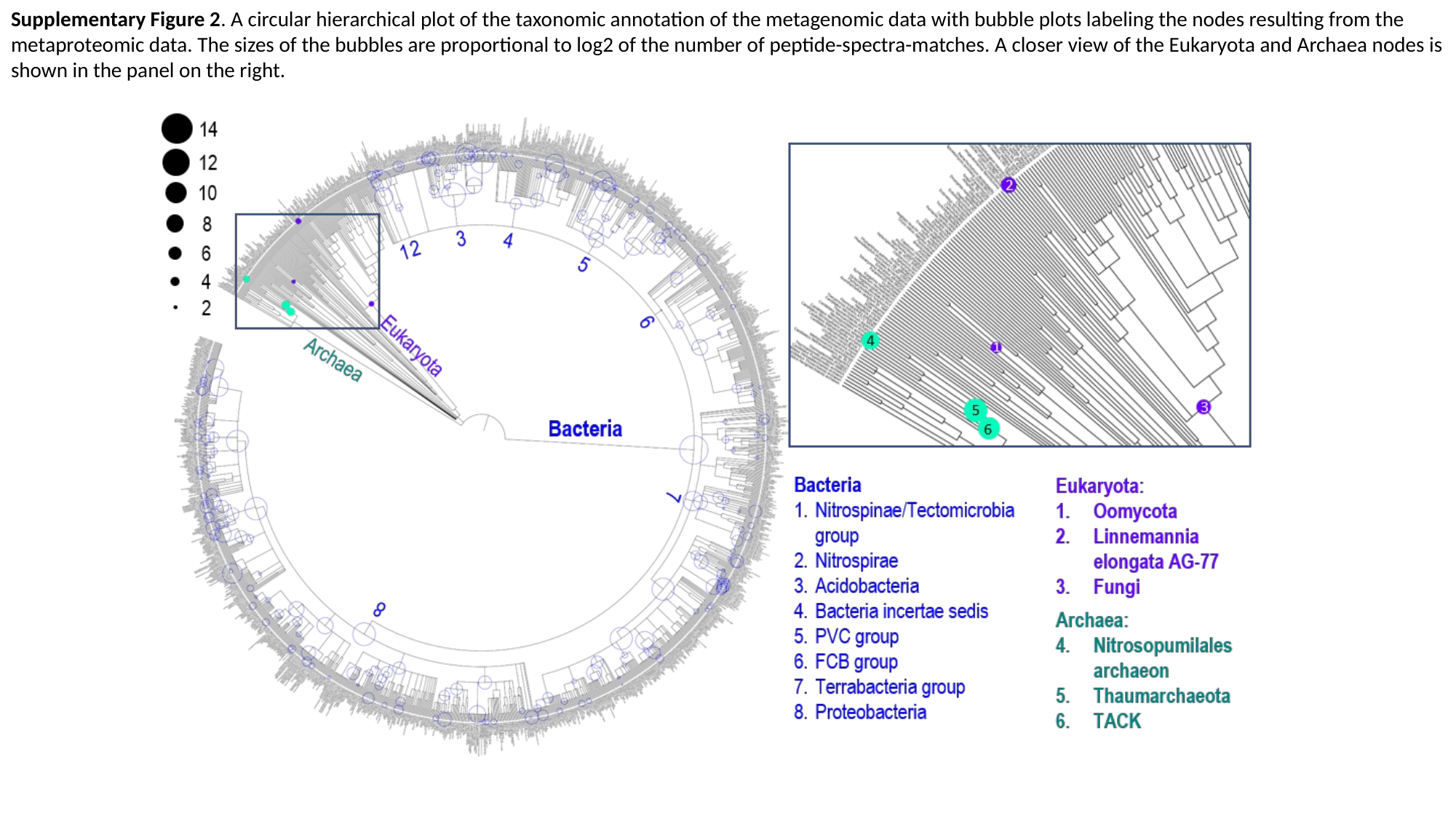

Supplementary Figure 2. A circular hierarchical plot of the taxonomic annotation of the metagenomic data with bubble plots labeling the nodes resulting from the metaproteomic data. The sizes of the bubbles are proportional to log2 of the number of peptide-spectra-matches. A closer view of the Eukaryota and Archaea nodes is shown in the panel on the right.

## Slide 3
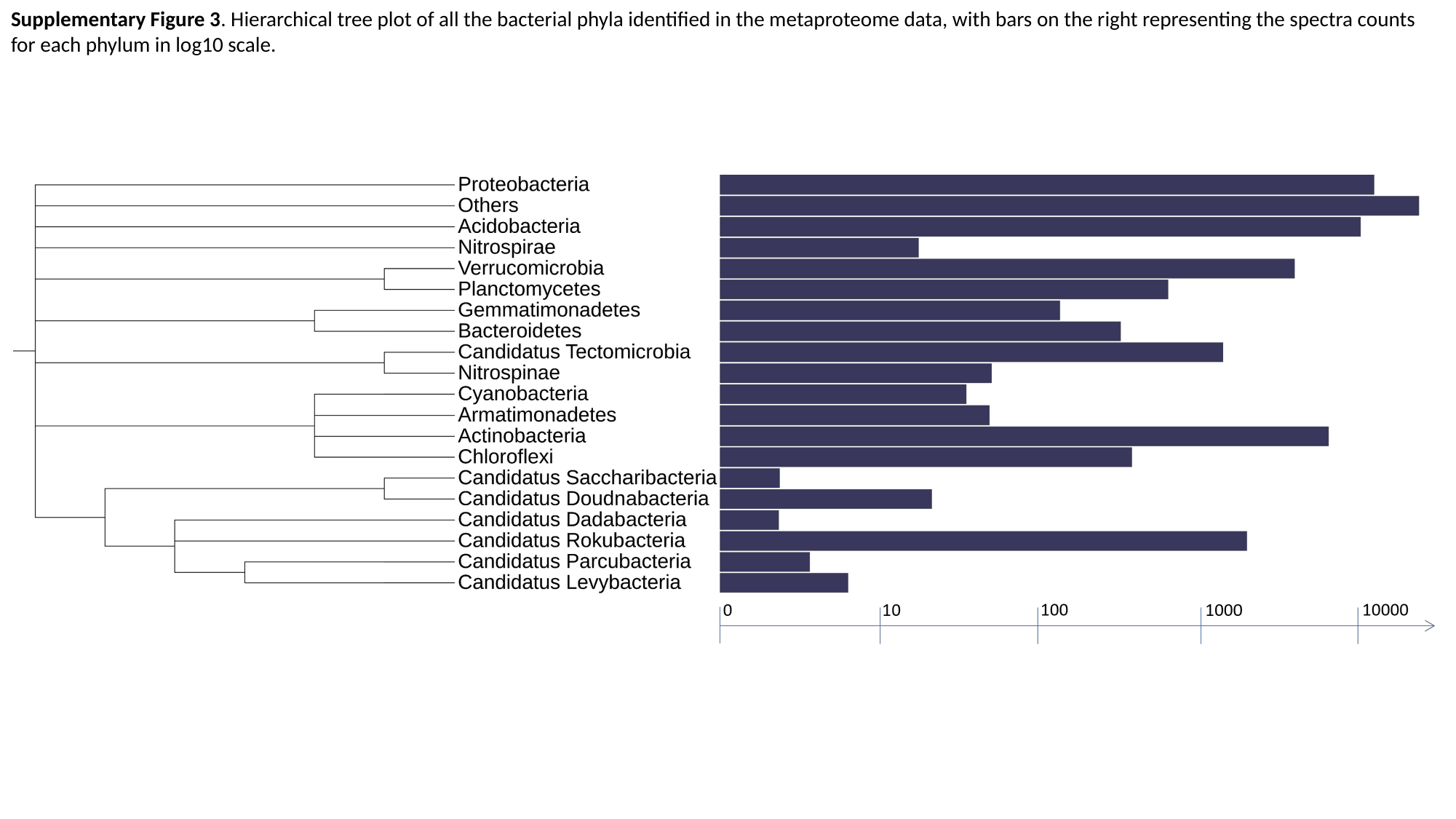

Supplementary Figure 3. Hierarchical tree plot of all the bacterial phyla identified in the metaproteome data, with bars on the right representing the spectra counts for each phylum in log10 scale.

## Slide 4
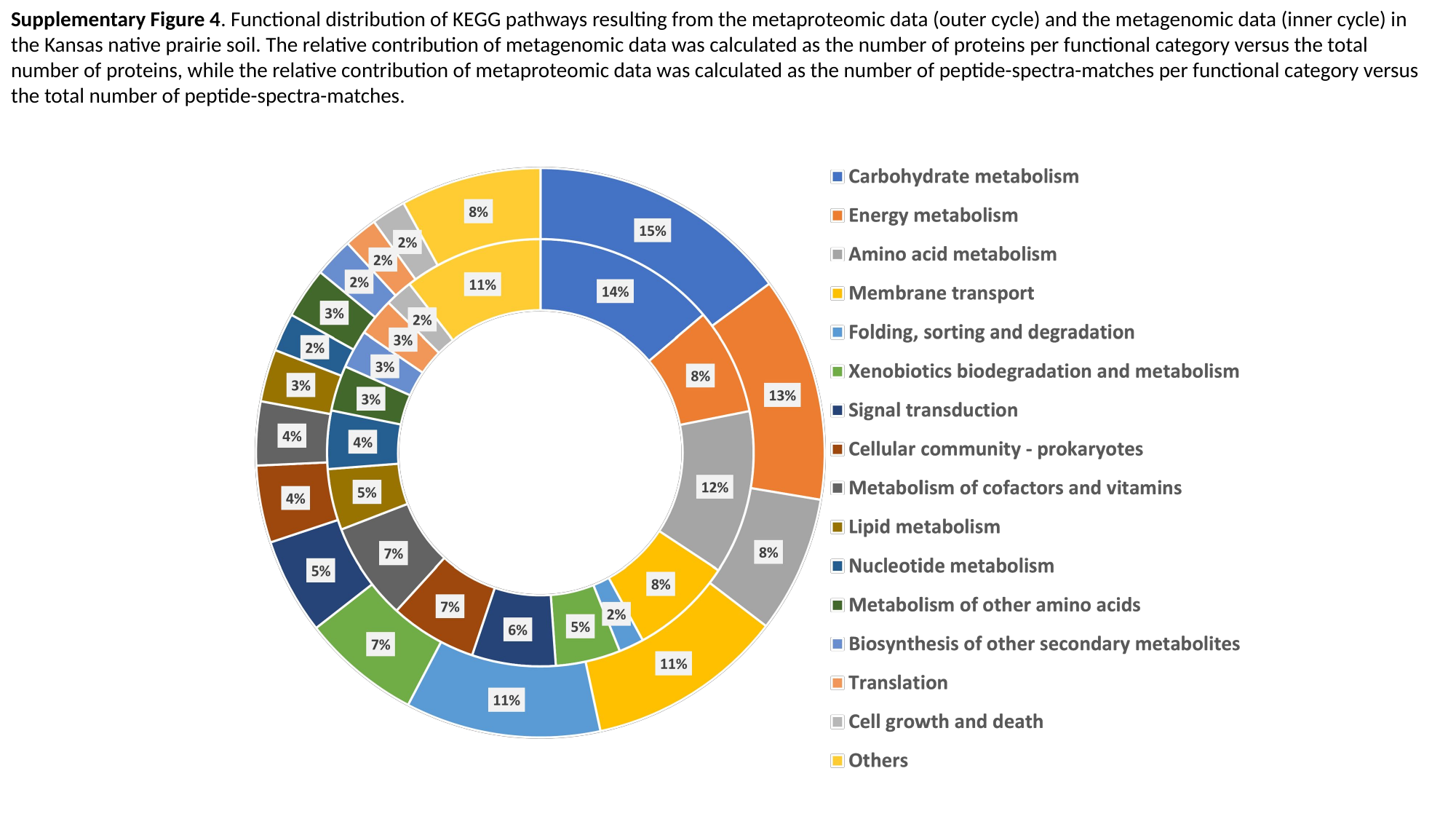

Supplementary Figure 4. Functional distribution of KEGG pathways resulting from the metaproteomic data (outer cycle) and the metagenomic data (inner cycle) in the Kansas native prairie soil. The relative contribution of metagenomic data was calculated as the number of proteins per functional category versus the total number of proteins, while the relative contribution of metaproteomic data was calculated as the number of peptide-spectra-matches per functional category versus the total number of peptide-spectra-matches.

## Slide 5
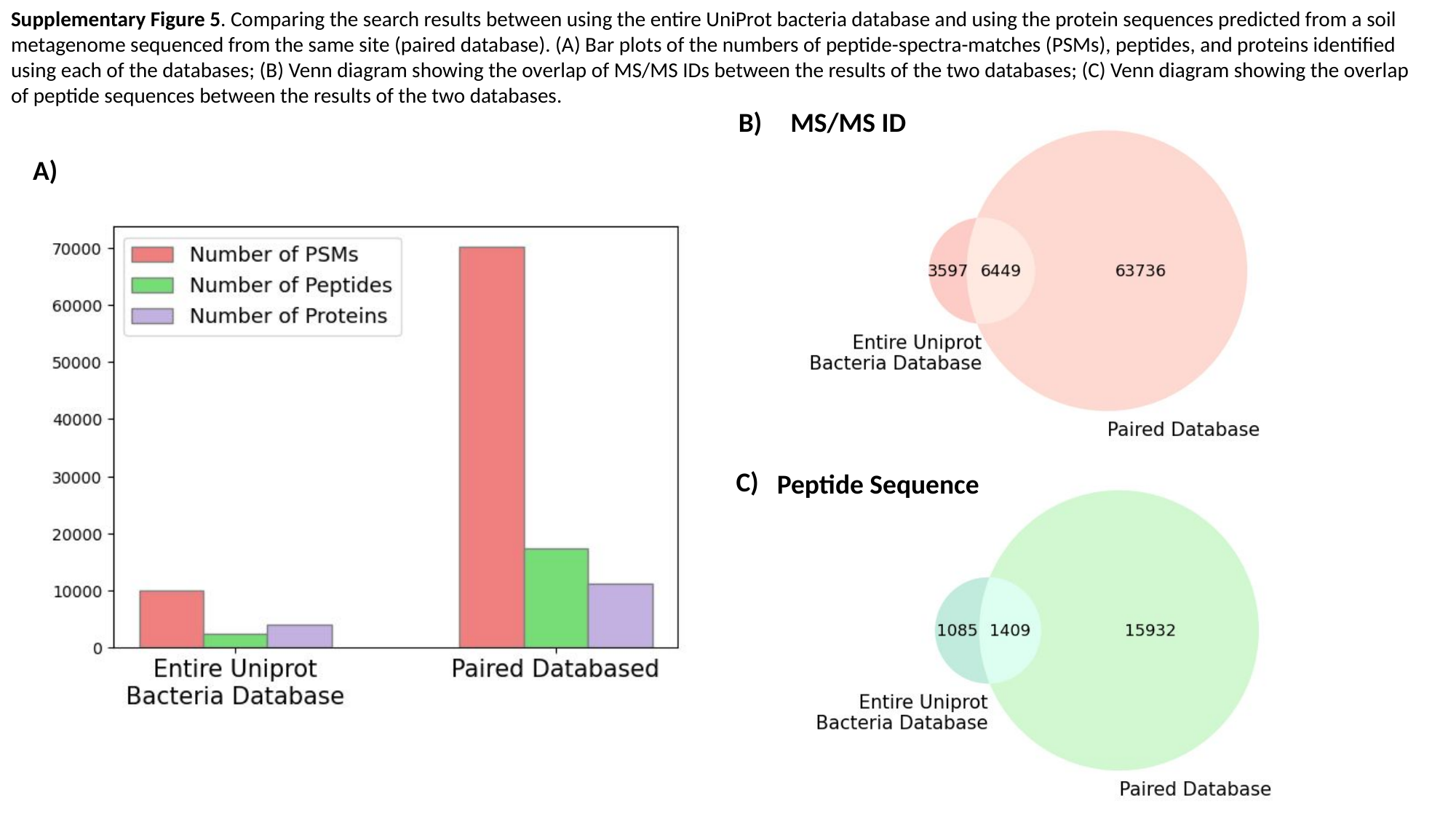

Supplementary Figure 5. Comparing the search results between using the entire UniProt bacteria database and using the protein sequences predicted from a soil metagenome sequenced from the same site (paired database). (A) Bar plots of the numbers of peptide-spectra-matches (PSMs), peptides, and proteins identified using each of the databases; (B) Venn diagram showing the overlap of MS/MS IDs between the results of the two databases; (C) Venn diagram showing the overlap of peptide sequences between the results of the two databases.
B)
MS/MS ID
A)
C)
Peptide Sequence

## Slide 6
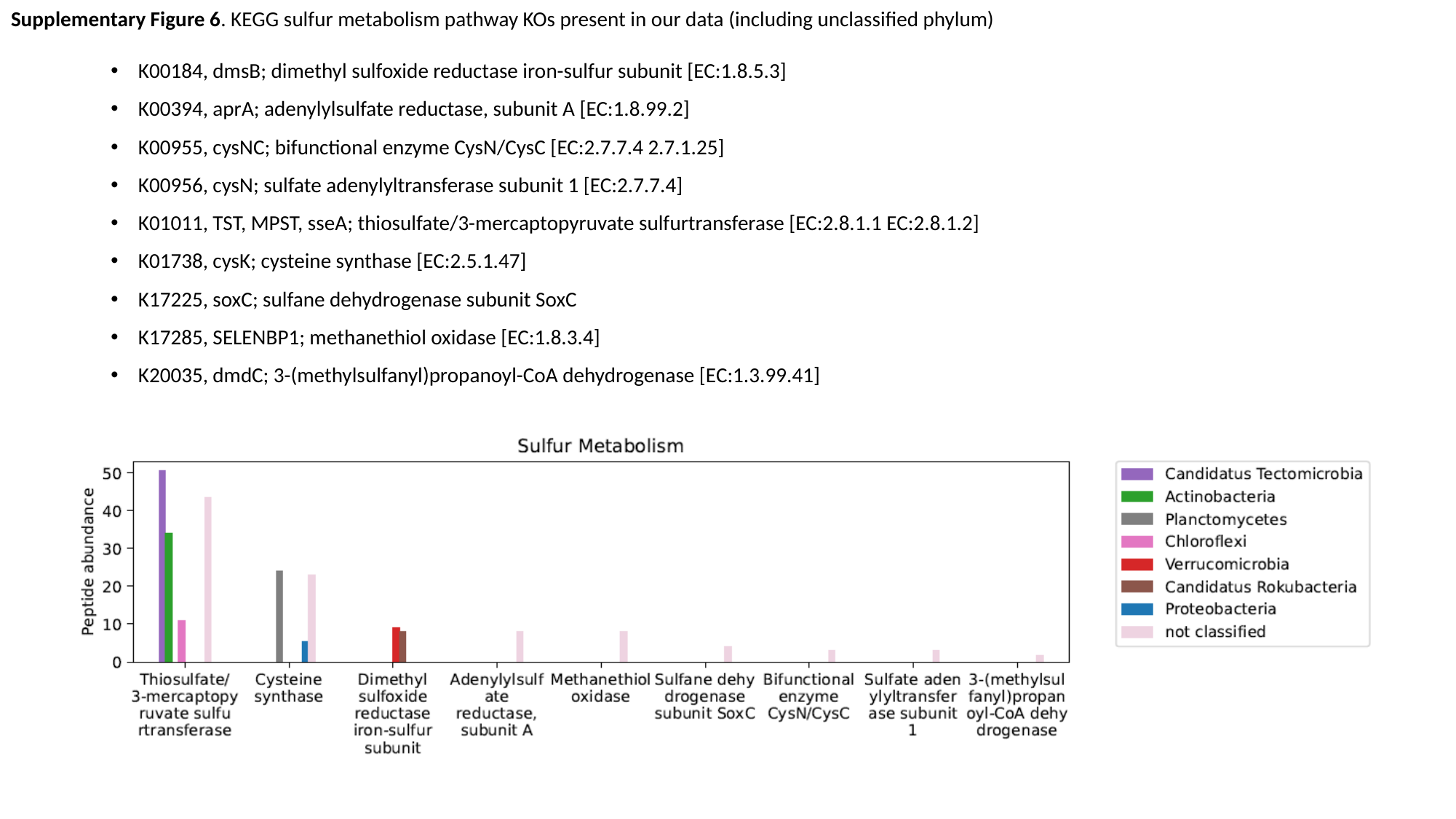

Supplementary Figure 6. KEGG sulfur metabolism pathway KOs present in our data (including unclassified phylum)
K00184, dmsB; dimethyl sulfoxide reductase iron-sulfur subunit [EC:1.8.5.3]
K00394, aprA; adenylylsulfate reductase, subunit A [EC:1.8.99.2]
K00955, cysNC; bifunctional enzyme CysN/CysC [EC:2.7.7.4 2.7.1.25]
K00956, cysN; sulfate adenylyltransferase subunit 1 [EC:2.7.7.4]
K01011, TST, MPST, sseA; thiosulfate/3-mercaptopyruvate sulfurtransferase [EC:2.8.1.1 EC:2.8.1.2]
K01738, cysK; cysteine synthase [EC:2.5.1.47]
K17225, soxC; sulfane dehydrogenase subunit SoxC
K17285, SELENBP1; methanethiol oxidase [EC:1.8.3.4]
K20035, dmdC; 3-(methylsulfanyl)propanoyl-CoA dehydrogenase [EC:1.3.99.41]

## Slide 7
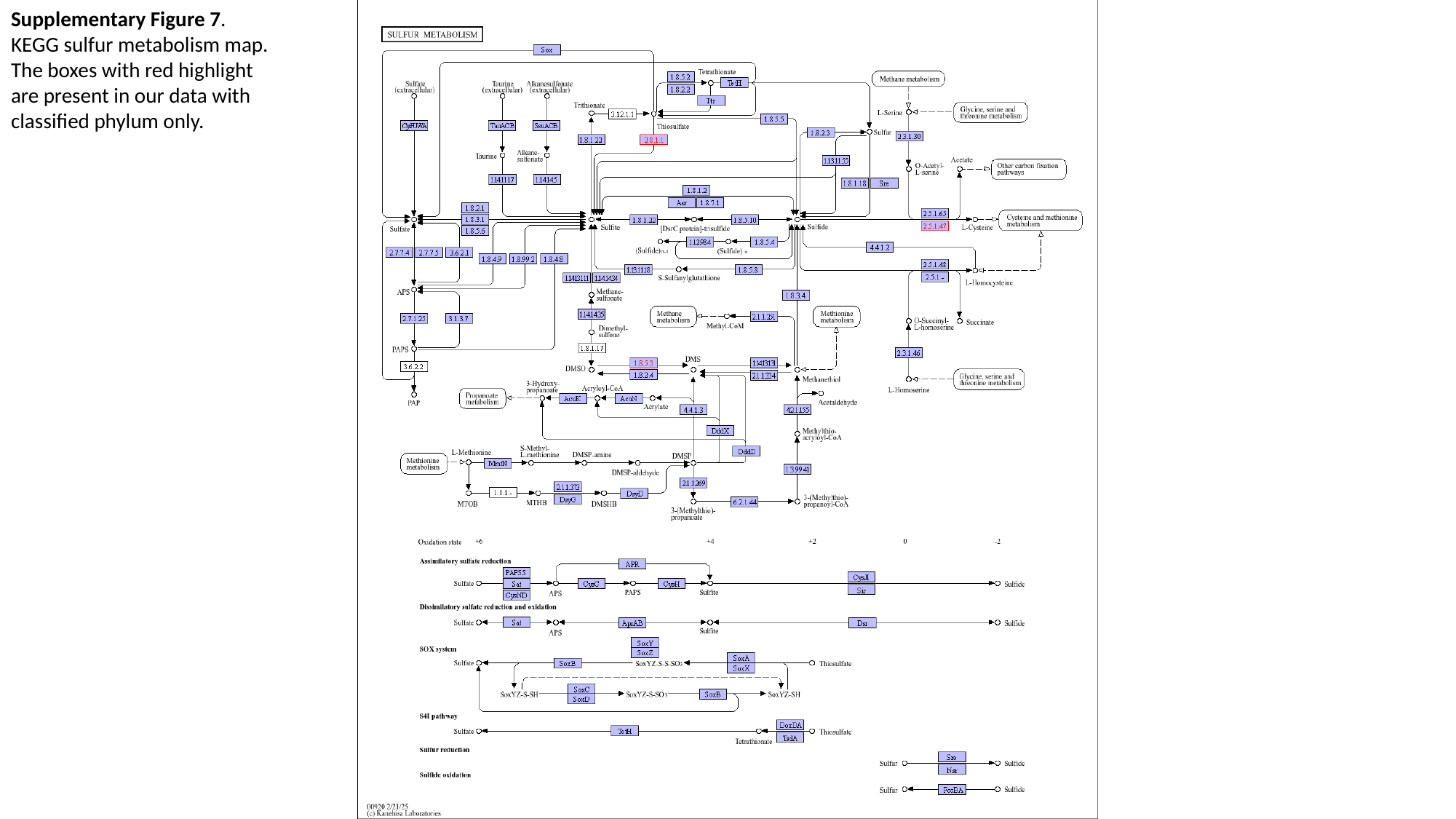

Supplementary Figure 7. KEGG sulfur metabolism map. The boxes with red highlight are present in our data with classified phylum only.

## Slide 8
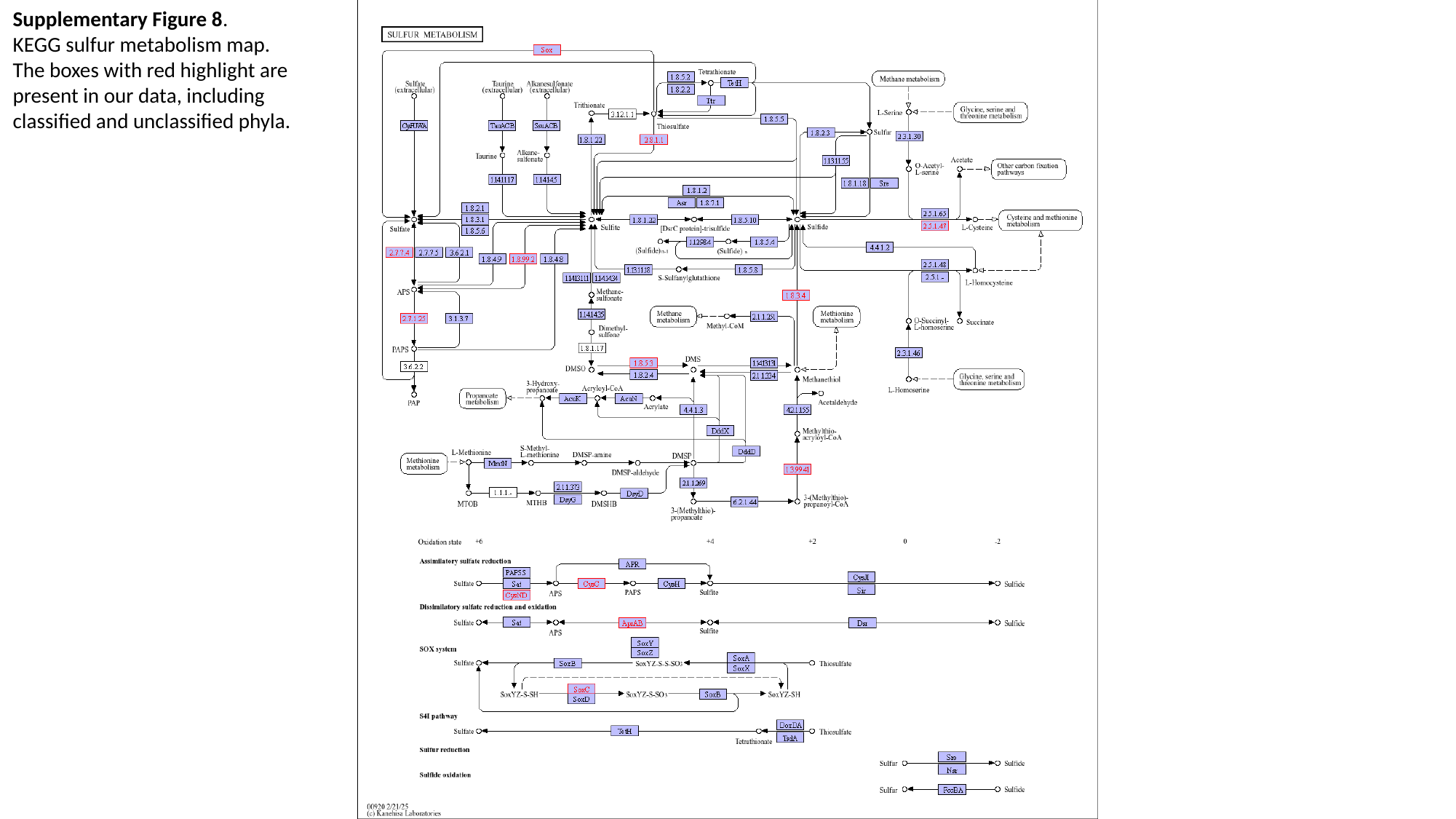

Supplementary Figure 8.
KEGG sulfur metabolism map.
The boxes with red highlight are present in our data, including classified and unclassified phyla.
